# Zoonotic Flavivirus Exposure in Peri-Urban and Suburban Pig-Keeping in Hanoi, Vietnam, and the Knowledge and Preventive Practices of Pig Farmers

**DOI:** 10.3390/tropicalmed7050079

**Published:** 2022-05-19

**Authors:** Long Pham-Thanh, Thang Nguyen-Tien, Ulf Magnusson, Vuong Nghia Bui, Anh Ngoc Bui, Åke Lundkvist, Duoc Trong Vu, Son Hai Tran, Minh Xuan Can, Hung Nguyen-Viet, Johanna F. Lindahl

**Affiliations:** 1 International Livestock Research Institute (ILRI), Hanoi 10000, Vietnam; thang.t.nguyen@cgiar.org (T.N.-T.); h.nguyen@cgiar.org (H.N.-V.); j.lindahl@cgiar.org (J.F.L.); 2 Department of Medical Biochemistry and Microbiology, Uppsala University, 75123 Uppsala, Sweden; ake.lundkvist@imbim.uu.se; 3 Department of Animal Health, Ministry of Agriculture and Rural Development, Hanoi 10000, Vietnam; 4 Department of Clinical Sciences, Swedish University of Agricultural Sciences, 75123 Uppsala, Sweden; ulf.magnusson@slu.se; 5 National Institute for Veterinary Research, Hanoi 10000, Vietnam; buinghiavuong@gmail.com (V.N.B.); buingocanh_1980@yahoo.com (A.N.B.); 6 National Institute for Hygiene and Epidemiology, Hanoi 10000, Vietnam; vu.duoc@gmail.com (D.T.V.); haison20284@yahoo.com (S.H.T.); 7 Hanoi Sub-Department of Livestock Production and Animal Health, Hanoi 10000, Vietnam; minhcx@gmail.com

**Keywords:** flaviviruses, knowledge and practices, pigs, serology, mosquitoes, risk factors, Hanoi

## Abstract

Mosquito-borne diseases (MBDs), including those caused by flaviviruses, remain human health problems for developing and urbanizing economies. This cross-sectional study examined risks of flavivirus exposure through a survey regarding knowledge and practices of pig farmers, and serological analysis of pigs in peri-urban and suburban Hanoi city. A total of 636 pig sera from 179 pig farms in 4 districts, namely, Chuong My, Dan Phuong, Ha Dong, and Bac Tu Liem, were analyzed by a competitive ELISA designed for flavivirus antibody detection. The results indicated a low level of awareness about MBDs among pig farmers, and a high seroprevalence in pigs at 88.5% (95%CI = 85.8–90.9%). Moreover, common practices of pig owners to prevent mosquitoes at home and farm did not show a significant reduction in flavivirus exposure in pigs. At animal level, significant associations between seropositive pigs and the farms with more than 60 pigs, and the district location were found. Farm-level multivariable analysis did not identify significant risk factors for flavivirus exposure. The study suggests that improving awareness of pig owners about MBDs in Hanoi city may be warranted to reduce the risk for MBD flavivirus infections in both humans and pigs.

## 1. Introduction

Mosquito-borne zoonotic flavivirus infections cause several million human cases annually [1]. Pigs, a domestic animal species, can be infected by several flaviviruses, such as Japanese encephalitis virus (JEV), dengue fever virus (DENV), Zika virus (ZIKV), and West Nile virus (WNV) [2,3,4,5]. Moreover, pigs are the main amplifying host of JEV infection [6] and constitute a potential source of Japanese encephalitis (JE) transmission to humans. Flaviviruses have a tendency to emerge, which is evident by the recent spread of JEV in Australia in 2022 [7], and there are fears that the virus will spread to other continents as competent *Culex* vectors are also present in Europe [8,9].

The rapid urbanization of Hanoi city provides suitable conditions for transmission of mosquito-borne diseases (MBDs), such as JE and dengue [10]. This is because there is a wide range and high density of hosts for blood-feeding and mosquito vectors, with 7.9 million inhabitants, 1.8 million pigs, 0.4 thousand horses, 136 thousand cattle, 23.5 thousand buffaloes, 11.5 thousand goats, 31.5 million poultry, and 450.3 thousand dogs, as of 2018 [11]. In addition, a large rice paddy area of suburban Hanoi, the most preferred habitat of *Culex* mosquito vectors [12], occupies 179,546 hectares in 2018 [11]. However, the understanding of mosquito-borne zoonotic flavivirus circulation in Hanoi remains limited.

In Vietnam, human vaccines against JE are available [13], and a dengue vaccination has been trialed [14], but no official vaccination programs have been applied in pigs. Controlling mosquito vectors is one of the most important measures to prevent many MBDs; nevertheless, the implementation of the control measure still lacks community engagement that strongly relies on local people’s knowledge and practices [15].

The objectives of the study were to assess the perception and preventive practices of pig farmers, and to investigate risk factors for mosquito-borne zoonotic flavivirus infections in pig-keeping households in suburban Hanoi.

## 2. Materials and Methods

### 2.1. Study Design

**Figure 1:**
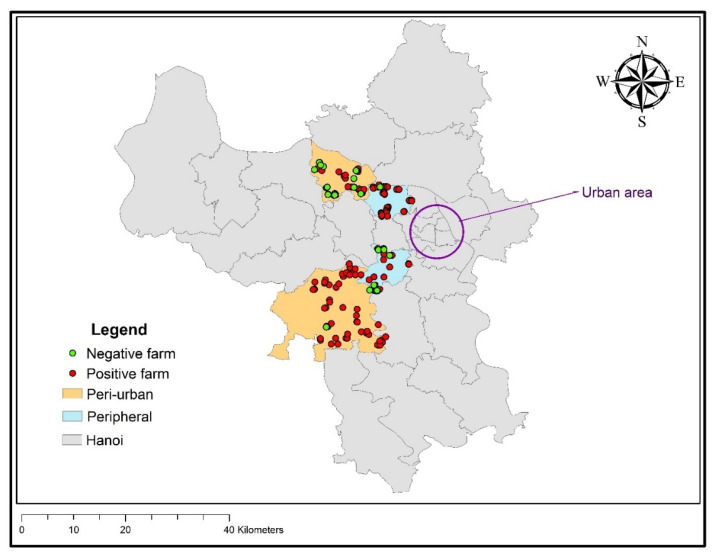
Distribution of sampled pig farms in Hanoi, Vietnam, and flavivirus serological results.

A cross-sectional study was implemented in pig farms of two suburban, peripheral city districts where less than 1000 cattle and buffaloes, 15,000 pigs, and 150,000 poultry per district are kept, and in two more peri-urban districts with more than 1000 large ruminants, 15,000 pigs, and 150,000 poultry of Hanoi, Vietnam, from September to October 2018 (Figure 1).

Multi-stage sampling strategy was used as described earlier [16]. In brief, random selection of 20 global positioning system (GPS) points was made in every selected district, and about three pig-keeping households within a radius of 2 km from each GPS point were visited. The sample size calculation was described in our previous study describing differences between households with and without livestock, but was, in brief, based on a power of 0.8, a significance level of 0.05, and a desired detection of 20% difference between households with livestock keeping compared to households without livestock keeping [16].

On each farm, the pig owner or the person taking care of the pig(s) was interviewed using a structured questionnaire form, and a maximum of five pigs were randomly selected for flavivirus serology testing based on the expected maximum that a farmer would allow.

### 2.2. Questionnaire and Interviews

The questionnaire was developed with three major sections: the respondent’s demographic characteristics (ten questions); information on pig as well as livestock keeping and animal diseases (thirteen questions); awareness (fourteen questions) that included knowledge about MBDs (with 22 scored items) and practices to prevent MBDs (with 11 scored items). The questionnaire was administered on pig farms through direct face-to-face interviews with pig keepers. Every appropriate answer to either knowledge or practice questions was awarded one point, while incorrect answers received a zero score.

### 2.3. Blood Sample Collection and Storage

Pig blood was collected from *Vena cava* by trained veterinarians of Hanoi Sub-Department of Livestock Production and Animal Health. The samples were stored in a cool box in the field and transferred to the National Institute for Veterinary Research (NIVR) within the sampling day, where sera were centrifuged and separated immediately, then they were kept at −20 °C until testing.

### 2.4. Mosquito Collection and Identification

Mosquito sampling was conducted by trained staff of NIVR using Centers for Disease Control and Prevention (CDC) light traps [17] and Biogents Sentinel (BG) traps [18]. A pair of traps were used on each farm, in which one CDC trap was hung outdoors close to the pig pen at about 1.5 m height above the ground; and another indoor trap (either CDC light trap or BG trap) was placed near the bedroom of pig owner. In the more rural settings, both BG and CDC traps have been found to provide a similar attraction to mosquito species [19]. The traps were activated from 5 p.m. on the interviewing day until 7.30 a.m. of the next day, the active phase of some species of the *Anopheles*, *Mansonia*, *Aedes*, and *Culex* genera [20]. The mosquitoes collected in each trap were transferred to a labeled 50 mL conical centrifuge tube using a battery-operated aspirator, kept in a cool box in the field, and sent to the National Institute of Hygiene and Epidemiology (NIHE) on the same day as mosquito collection. Mosquito samples were stored at −80 °C in laboratory until identifying sex and species of the mosquitoes at NIHE. Only female mosquitoes were recorded for analysis.

### 2.5. Laboratory Technique

The serum samples were tested by a competitive enzyme-linked immunoassay (cELISA) kit for detection of IgG antibodies against WNV manufactured by IDvet company (No. 310, rue Louis Pasteur, Grabels, France). This kit has been shown to allow for detection of antibodies against several flaviviruses of different animal species [21,22]. In principle, samples to be tested, and controls, were added to the precoated plate with the pr-E protein of WNV, which includes epitopes common to several flaviviruses such as WNV, JEV, and DENV. Anti-pr-E antibodies in a serum sample form an antigen–antibody complex. An anti-pr-E antibody HRP conjugate binds to the remaining free pr-E epitopes, forming an antigen–conjugate–peroxidase complex. Each pig serum was tested in duplication in the same ELISA plate. Calculation of percentage inhibition (S/N%) was equal to the mean optical density (OD) value of sample divided by the mean OD value of negative control, multiplied by 100. Samples presenting a Sample/Negative control quota less than or equal to 40% were considered positive; higher than 40% and less than or equal to 50% were considered doubtful; higher than 50% were considered negative. Doubtful results were excluded from analyses.

### 2.6. Statistical Analysis

In the four districts, 192 pig farms were visited, and 704 blood samples were tested by the cELISA. However, 13 pig farms missed information on knowledge and practices, and 53 serum samples from these 13 farms and 15 serum samples of other farms provided doubtful results by the cELISA and were therefore excluded from the analysis. Finally, a total of 179 farms and 636 pigs were analyzed. A positive farm was defined as having at least one pig seropositive by the cELISA. The presence of mosquito vectors, which were handled both as a binary variable and the total number of vectors in each farm, was summed.

Data obtained from the questionnaires and the laboratory results were recorded as variables in Excel^®®^ spreadsheets and transferred into STATA/SE 15.0 (StataCorp LLC, College Station, TX, USA) for analysis.

Social-demographic characteristics, and a summary of knowledge and practices of pig raisers were expressed in percentages. Spearman’s rank correlation analysis was used to determine the relationship between knowledge and practices. The Mann–Whitney U test, Kruskal–Wallis test, and negative binomial regression were used to identify associations between demographic factors and knowledge and practice scores. Variables with a *p*-value less than 0.2 in univariable analysis were included in the multivariable negative binomial regression.

Mosquito species classified as vectors of MBDs were summarized in quantity and proportions. The difference in mosquito numbers collected indoor and outdoor on each farm was calculated, and a mean difference in the two subpopulations of indoor and outdoor mosquitoes for all pig farms was determined by the paired *t*-test.

For risk factor identification for seropositivity of pigs against flaviviruses, the association of independent variables and outcomes was evaluated using a chi-square test in univariable analysis. At farm level, the correlation of all independent variables was assessed. Variables in univariable analysis that provided a *p*-value lower than 0.25 [23] and absolute correlation coefficient value (r-value) below 0.7 [24] were applied in logistic regression models. Variables changing more than 25% of coefficients of other variables were classified as confounding factors and they were moved back to the model if the affected variables were significant. Mixed-effects multivariable logistic regression models were built using meqrlogit in STATA/SE 15.0 with pig-keeping household as a random effect for the animal level model, while the district was a random effect for the farm level model. A stepwise backward manual elimination was performed to identify confounders and significant predictors in the model. A *p*-value of less than 0.05 was considered statistically significant.

## 3. Results

### 3.1. Pig Farmer Demographics

Of the 179 respondents, the male gender accounted for 75.4% of the surveyed population. Most participants (67.3%) were aged between 40 and 59 years old; the mean age was 50.35 ± 9.50. Over half of the participants (54.4%) had graduated secondary school, followed by a high-school level (29.4%), and some has obtained college or university degrees (6.7%). Most respondents (80.9%) worked with farming as their main job, and 85.5% of pig farms kept less than 60 pigs (Table 1).

### 3.2. Knowledge of Pig Farmers

Most respondents were aware of dengue fever (93.9%), followed by malaria (61.5%) and Zika (37.4%), but only a few respondents had heard about Japanese encephalitis (7.8%) and filariasis (2.8%) (Table 2). About 3.9% of participants were not aware of any MBD. More than 73.3% of participants recognized polluted water, stagnant water containers, or water tanks as suitable breeding sites of mosquitoes. About 30% of respondents listed discarded car tires, flower vases, or bonsai rockery as potential mosquito breeding sites. Few participants (1.7%) did not know any breeding sites of mosquitoes. Most of the participants (76.5%) considered warm and humid weather as a risk factor for contracting MBDs, while some of them (40.2%) considered livestock keeping as important. Participants listed symptoms of patients contracting MBDs, which included high fever (89.4%), hemorrhage (56.4%), severe headache (39.7%), nausea or vomiting (30.2%), muscle pain (28.5%), and rash (17.9%).

### 3.3. Practices of Pig Farmers

The MBD preventive practices of pig farmers at home and farm are summarized in Table 3. The most common preventive measures that respondents applied against mosquito biting were bed nets (94.4%), followed by mosquito electric rackets (53.1%) and insecticides (52%). Other mosquito control methods were also used including covering water tanks with lids (34.1%), keeping fish in water containers (33%), eliminating breeding sites (30.2%), and wearing long-sleeve clothes (25.1%). A few individuals applied mosquito coil burning, repellents, larvicides, or window/door screening to prevent mosquito bites. 

### 3.4. Associations between Knowledge and Practice and Demographics

The median scores with IQR for knowledge and practices of pig farmers in Hanoi were 9 ± 5 (ranging from 0 to 22) and 3 ± 2 (ranging from 0 to 11), respectively. Spearman’s rank correlation analysis showed a strong positive correlation between knowledge and practices (Spearman’s rho = 0.68, *p* < 0.001).

Table 4 presents associations between the demographic factors and the knowledge and practices of pig farmers. District location and university education were significantly associated with both knowledge and practices (*p* < 0.05). The pig farmers in the districts of Bac Tu Liem and Dan Phuong showed significantly higher scores for their knowledge and practices than those in Chuong My district. However, the farmers in Ha Dong district had significantly higher scores for knowledge, but not a significant difference in the practice scores compared to the pig keepers in Chuong My district. The farmers who had graduated from a university obtained knowledge scores significantly higher than those at the primary level of education, but the practice score was not significantly different between higher education levels and the primary level. There were no significant differences in the knowledge and practice scores of the high school and secondary school levels, as compared to the primary level. Gender, age, occupation, and experience of a family member with MBDs (either dengue fever, JE, zika, malaria, or filariasis) were not found to be associated with knowledge and practice in the multivariable negative binomial regression analysis.

### 3.5. Mosquito Vectors

Of the 179 farms surveyed, a total of 88% had the presence of potential mosquito vectors (*Culex, Aedes, Mansonia, Armigeres,* and *Anopheles*), with 100%, 85%, 85%, and 85% of pig farms having vector mosquitoes in the Ha Dong, Bac Tu Liem, Chuong My, and Dan Phuong districts, respectively (Table 5).

A total of 7699 mosquitoes were collected and classified into the five different genera, of which 3039 mosquitoes were from the bedroom area defined as indoor mosquitoes and 4660 mosquitoes collected at the pig pen were defined as outdoor mosquitoes. Among the indoor mosquitos, the *Culex* mosquitoes were dominant with more than 94.5%, as compared with other genera of *Aedes, Mansonia, Armigeres,* and *Anopheles*. Of the total genera, *Cx. tritaeniorhynchus* constituted 74.6%, followed by the *Cx. vishnui* sub-group (8.7%), *Cx. quinquefasciatus* (6.5%), and *Cx. gelidus* (4.6%) (Table 6). Similarly, the species of *Cx. tritaeniorhynchus*, *Cx. gelidus,* and *Cx. vishnui* around pig pens were found at 66.5%, 13.6%, and 8.4%, respectively (Table 7). The numbers of indoor versus outdoor mosquitoes were not significantly different (*p* = 0.202) (Table 8).

### 3.6. Univariable Analysis at Animal Level

A total of 636 pigs from 179 farms in the four districts of suburban Hanoi were examined for antibodies against flaviviruses by a cELISA kit. Apparent seroprevalence was 88.5% (95% CI = 85.8–90.9%). Seroprevalence of pigs of smallholders with less than 10 animals (71.4%; 95% CI = 62.4–80.4%) was significantly lower than small farms of less than 30 pigs (89.1%; 95% CI = 85.2–92.9%), and small-medium farms of less than 60 pigs (94.2%; 95% CI = 90.8–97.5%), and medium farms with more than 60 pigs (93.5%; 95% CI = 88.5–98.6%). The seroprevalence of pigs under 4 months old varied from 90.1% for 4 months old pigs to 98.2% for 2 months old, which was significantly higher than for slightly older pigs, above 6 months old, at 65.9% (*p*-value < 0.05).

The seropositivity found in the peri-urban district Dan Phuong (68.3%; 95% CI = 60.0–76.7%) was significantly lower than in the peripheral districts of Ha Dong (89.7%; 95% CI = 83.6–95.8%), Bac Tu Liem (92.6%; 95% CI = 83.6–95.8%), and the Chuong My peri-urban district (95.8%; 95% CI = 92.9–98.6%).

Univariable analyses at animal level (Table 9) determined significant associations between seropositive pigs and herd size, age of pig, and district location.

### 3.7. Multivariable Analysis Results at Animal Level

The mixed-effects multivariable logistic regression model identified significant associations between the seropositivity of pigs and the larger farm size with at least 60 pigs, and the location of Ha Dong and Chuong My districts (*p* < 0.05), while the breed and the age were not a risk factor (Table 10).

### 3.8. Univariable Analysis Results at Farm Level

A positive farm was defined as having at least one pig positive by the cELISA test. The results of univariable analyses at farm level identified significant associations between seropositive farms and herd size; some mosquito control measures of the owners consisted of using repellent, wearing long-sleeve clothes, and covering the lid on water tanks (Table 11).

The odds of being seropositive in the pig herds with 10 to 29 pigs were significantly higher than in the herds of less than 10 pigs (OR = 7.85, *p* = 0.003). Pig farms where the owner was not using repellents had a higher odds ratio of being seropositive (OR = 6.22, *p* = 0.008). A similar higher seropositivity at pig farms was recorded by not wearing long-sleeve clothes by pig keepers (OR = 6.05, *p* = 0.009), and not closing the lid of the water tank (OR = 7.58, *p* = 0.01).

There was no significant difference in seropositivity of farms depending on district location, and mosquito vectors. Likewise, significant associations between seropositivity and mosquito-borne disease history in the family, mosquito prevention by window/door screening, electric trap or racket, mosquito coil burning, larvicides, spraying insecticide, and keeping fish inside water tanks were not found in this study.

### 3.9. Multivariable Analysis Results at Farm Level

The multivariable analysis showed a significant positive association between the seropositivity of pig farms and the practice of “breeding site elimination” (Coefficients =−6.61; *p* = 0.021), with more farms eliminating breeding grounds having a higher risk for positive animals, whereas the other variables were not significantly associated with the seropositivity of farms.

## 4. Discussion

The results of this study illustrated gaps in pig farmer awareness about MBDs, as well as their preventive practices. In particular, the knowledge score was 9 out of 22, and the practice score was 2 from the total of 11. Pig keepers with university education depicted better knowledge and practices as compared to the primary level (*p* < 0.05), which is in agreement with other studies aiming to support MBDs control for lower educational populations [25,26]. Most of the farmers of Hanoi knew that dengue and malaria are caused by mosquito vectors. In contrast, few of them (7%) mentioned JE, which is much lower as compared to a previous study in Kathmandu (42%) and Morang (25%) of Nepal [27]. At least 6% of the respondents were not able to mention any potential risk factors of contracting MBDs as well as any symptoms, while most of the remaining participants only mentioned high fever or/and hemorrhage. About 40% of the farmers did not recognize livestock playing a role in MBD transmission. This finding is similar to a previous study in India [28]. The pig farmers from different occupational backgrounds did not have a significant difference in their awareness.

Typical characteristics of smallholder pig farms in Hanoi are that most of the pig pens are constructed inside the family garden close to the owner’s house and many pig owners prefer to keep several species such as cattle, poultry, and pets on their farm. Taking into account that an average flight distance of mosquito species can be from several hundred meters for *Aedes* mosquitoes [29,30] to several kilometers for *Culex* genus [31], gives the opportunity for MBD circulation among human, livestock, and vector populations in suburban Hanoi.

Our study identified that *Culex* mosquitoes, especially *Cx. tritaeniorhynchus*, were prominent in the pig farms in Hanoi. This finding is similar to a study in China, showing that 73% of the mosquito abundance at pig farms was *Cx. tritaeniorhynchus* [32]. A previous study demonstrated that *Cx. tritaeniorhynchus* was found at 69%, followed by *Cx. vishnui* at 19% in Hanoi, as of 2004 [33]. Several *Culex* species including *Cx. quinquefasciatus, Cx. vishnui* subgroup, *Cx. gelidus, Cx. fuscocephalus,* and other genera of *Aedes, Mansonia, Armigeres,* and *Anopheles* were also present in pig rearing areas in suburban Hanoi.

Some studies have demonstrated a strong correlation between vector abundance and seropositive against JEV, a zoonotic flavivirus, in pigs [34,35]. The flavivirus seropositivity in the pig population was 88.5%, which was much higher than a previous study that used the same ELISA kit in northern Vietnam at 60.4% [36], which could be explained by the fact that our study also included younger pigs still having maternal antibodies. We recorded a gradual decline in seropositivity from 98.2% of two months old pigs, the age that maternal antibodies start disappearing [37], to 65.9% of pigs older than 6 months. Further immunological investigation of flavivirus infections in pigs under field condition is suggested.

Our study indicated common mosquito control measures applied by pig farmers in Hanoi could not reduce seropositivity against flaviviruses in their pig herds. In fact, mosquito vector control can only work effectively in certain conditions such as having a good understanding of the mosquito vectors in the control area, sustainability of mosquito preventive practices, and maintenance of political support in controlling MBDs [38,39,40,41].

Multivariable analysis at animal level determined the pig farms with more than 60 pigs and the district location as major risk factors for seropositivity of flavivirus infections. However, at farm level, not eliminating mosquito breeding sites through other means was associated with significantly reduced seropositivity on pig farms. This is an unusual result that could be affected by confounding factors on the pig farms, because this elimination of mosquito breeding sites can break down the life cycle of mosquito vectors. It may in fact be that farms with a very high burden of mosquitoes are the ones that have both high seroprevalence, as well as the ones trying to eliminate breeding sites.

Vaccination against mosquito-borne flaviviruses is not practical for the large pig population, and thus serological monitoring in pigs could be useful to better understand the potential risks and epidemiology of flavivirus infections in pig farms.

Our study had some limitations. The pig owners allowed us to take samples from pigs at 2 to 18 months old, only; therefore, analyses for older pigs were missing. The survey was conducted in only two months in the dry season; however, mosquito trapping and pig blood sampling extension to different seasons in a whole year would provide a better epidemiological understanding of MBD flavivirus circulation in Hanoi city. Mosquito vector control practices of the pig farmers were not observed, just reported, and the effectiveness of mosquito control measures was not evaluated. The study acknowledges the limitation of missing full plaque reduction neutralization test (PRNT) analysis for specific antibodies against JEV, DENV, and ZIKV, which are the flaviviruses that are endemically circulating in Vietnam, and for all pig blood samples, due to limited budget of the project and the low diagnostic laboratory capacity in Vietnam.

## 5. Conclusions

The study indicates gaps in pig farmer perception and practices on MBD prevention, but a high seroprevalence for flaviviruses among the pig populations. In Hanoi, where pigs are kept close to human living areas, a high flavivirus seroprevalence in pig populations (88.5%) indicated a potential risk of mosquito-borne zoonotic flavivirus exposure to the inhabitants. Therefore, the need for awareness improvement for pig-keeping households is highlighted.

The *Culex* genus, mainly *Cx. tritaeniorhynchus*, was the most abundant mosquito in the pig farms of Hanoi. Furthermore, some common mosquito preventive measures of pig farmers did not significantly reduce seropositivity in their pigs. Our study could not find an association between the age of pigs and seropositivity in pigs; although, we found significant associations with the farms keeping at least 60 pigs and the district location.

## Figures and Tables

**Table 1:** Social-demographic features of pig farmers.

Characteristic	*n* (%)	Mean ± SD
**Gender**		
Male	135 (75.4)	
Female	44 (24.6)	
**Age**		50.35 ± 9.50
30–39	21 (13.2)	35.43 ± 2.56
40–49	49 (30.8)	44.12 ± 2.60
50–59	58 (36.5)	53.79 ± 2.92
60+	31 (19.5)	63.87 ± 3.57
**Education**		
Primary school	17 (9.4)	
Secondary school	98 (54.4)	
High school	53 (29.4)	
College/university	12 (6.7)	
**Occupation**		
Officer	6 (3.4)	
Farmer	144 (80.9)	
Unemployed	9 (5.1)	
Retired	7 (3.9)	
Others	12 (6.7)	
**Marriage status**		
Single	2 (1.1)	
Married	174 (97.8)	
Separated/Divorced	1 (0.6)	
Widowed	1 (0.6)	
**Pig herd size: Number of pigs per farm**		44.33 ± 99.92
<10	35 (19.5)	5.57 ± 2.19
10–29	71 (39.7)	16.94 ± 5.82
30–59	47 (26.3)	39.7 ± 7.42
≥60	26 (14.5)	179.6 ± 218.1

**Table 2:** Knowledge of pig farmers about mosquito-borne diseases.

Questions		
**Have you heard about diseases being transmitted from mosquitoes to humans?**	**N = 179**	**%**

No	7	3.9
Dengue fever	168	93.9
Japanese Encephalitis	14	7.8
Zika	67	37.4
Malaria	110	61.5
Filariasis	5	2.8
**Can you list breeding sites of mosquitoes?**		
Don’t know	3	1.7
Clean water	29	16.2
Drain/polluted water	143	79.9
Stagnant water containers	146	81.6
Car tires	55	30.7
Water tanks	132	73.7
Vase	60	33.5
Bonsai rockery	57	31.8
**Can you list the risk factors for getting mosquito-borne diseases?**		
Don’t know	11	6.1
Warm and humid season	137	76.5
High population density	47	26.3
Stagnant water containers	123	68.7
Livestock keeping	72	40.2
**Can you list any symptoms of mosquito-borne diseases?**		
Don’t know	13	7.3
High fever	160	89.4
Muscle pain	51	28.5
Nausea/vomiting	54	30.2
Severe headache	71	39.7
Rash	32	17.9
Hemorrhage	101	56.4

**Table 3:** Practices of pig farmers about mosquito-borne diseases.

Question: What Do You Do to Prevent Mosquito Bites?		
	N = 179	%
Don’t know	0	0
Screening of windows/doors	13	7.3
Mosquito repellent	22	12.3
Mosquito bed nets	169	94.4
Electric rackets	95	53.1
Coil burning	37	20.7
Long-sleeve clothes	45	25.1
Lidded the water tank	61	34.1
Chemical treatment in water containers	6	3.4
Anti-mosquito products/insecticides	93	52.0
Mosquito breeding site elimination	54	30.2
Fish keeping in water containers	59	33.0

**Table 4:** Knowledge (K) and practice (*p*) scores with respect to demographics.

Knowledge and Practice Scores with Respect to Demographics (N = 179)						
Variable	K-Score (Median ± IQR)	*p*-Value (Univariable)	*p*-Value (Multivariable)	*p*-Score (Median ± IQR)	*p*-Value (Univariable)	*p*-Value (Multivariable)
	9 ± 5			3 ± 2		
Gender *						
Male	9 ± 5	0.715	-	3 ± 2	0.506	
Female	10 ± 6			3 ± 2.5		
Family member experienced with MBD *						
Yes	9 ± 4	0.741	-	3 ± 0.5	0.757	
No	10 ± 5			3 ± 2		
District **						
Chuong My	7 ± 4	<0.001	Ref.	2 ± 1	<0.001	Ref.
Dan Phuong	13 ± 10		<0.001	6 ± 6		<0.001
Bac Tu Liem	12 ± 4		<0.001	3 ± 1		<0.001
Ha Dong	9 ± 2		0.038	3 ± 1		0.206
Age **						
30–39	9 ± 3	0.923	-	2 ± 1	0.224	-
40–49	9 ± 5			3 ± 2		
49–50	9 ± 4.5			3 ± 2		
60+	9 ± 5			3 ± 2		
Level of education **						
Primary school	10 ± 8	0.014	Ref.	3 ± 2.5	0.211	Ref.
Secondary	9 ± 5		0.670	3 ± 2		0.280
High school	9 ± 4		0.453	3 ± 2		0.098
College/University	12.5 ± 7.5		0.007	3.5 ± 5		0.058
Occupation**						
Officer	10.5 ± 3	0.037	Ref.	3 ± 5	0.038	Ref.
Farmer	10 ± 5.5		0.394	3 ± 2		0.913
Unemployed	6.5 ± 3.5		0.333	2 ± 0.5		0.163
Retired	12 ± 11		0.583	7 ± 5		0.725
Others	9 ± 2		0.909	3 ± 1		0.797

* Mann–Whitney U test; ** Kruskal–Wallis test; Ref., Reference.

**Table 5:** Summary of mosquito vector presence.

District	No. Pig Farm Surveyed	No. of Farm with Mosquito Vectors	%
Chuong My ^b^	53	45	85
Dan Phuong ^b^	41	35	85
Bac Tu Liem ^a^	53	45	85
Ha Dong ^a^	32	32	100
Total	179	157	88

Abbreviations: a, peripheral; b, peri-urban.

**Table 6:** Summary of mosquito species collected at bedroom—indoor.

Mosquito Species	Bac Tu Liem ^a^		Chuong My ^b^		Dan Phuong ^b^		Ha Dong ^a^		Total	
	No.	%	No.	%	No.	%	No.	%	No.	%
*Aedes albopictus*	6	0.9	5	1.9	6	1.6	1	0.1	18	0.6
*Culex tritaeniorhynchus*	479	69.8	181	68.8	224	58.8	1383	80.9	2267	74.6
*Cx. vishnui* subgroup	68	9.9	16	6.1	86	22.6	95	5.6	265	8.7
*Cx. quinquefasciatus*	70	10.2	18	6.8	19	5.0	90	5.3	197	6.5
*Cx. gelidus*	38	5.5	17	6.5	8	2.1	78	4.6	141	4.6
*Cx. fuscocephalus*	0	0.0	2	0.8	0	0.0	0	0.0	2	0.1
*Mansonia* spp.	0	0.0	1	0.4	0	0.0	0	0.0	1	0.0
*Ma. uniformis*	4	0.6	0	0.0	2	0.5	1	0.1	7	0.2
*Ma. annulifera*	3	0.4	1	0.4	12	3.1	1	0.1	17	0.6
*Ma. indiana*	0	0.0	0	0.0	0	0.0	1	0.1	1	0.0
*Armigeres* spp.	0	0.0	1	0.4	4	1.0	39	2.3	44	1.4
*Anopheles* spp.	0	0.0	0	0.0	0	0.0	1	0.1	1	0.0
*Anopheles aconitus*	0	0.0	0	0.0	0	0.0	1	0.1	1	0.0
*An. hycanus*	18	2.6	21	8.0	20	5.2	18	1.1	77	2.5
Total	686	100	263	100	381	100	1709	100	3039	100

Abbreviations: a, peripheral city district; b, peri-urban.

**Table 7:** Summary of mosquito species collected at pig pen—outdoor.

Mosquito Species	Bac Tu Liem ^a^		Chuong My ^b^		Dan Phuong ^b^		Ha Dong ^a^		Total	
	No.	%	No.	%	No.	%	No.	%	No.	%
*Aedes* spp.	0	0.0	1	0.2	1	0.1	0	0.0	2	0.0
*Aedes albopictus*	3	0.4	1	0.2	1	0.1	1	0.0	6	0.1
*Culex tritaeniorhynchus*	477	65.9	293	54.1	1057	80.7	1306	62.6	3175	66.5
*Cx. vishnui* subgroup	116	16.0	3	0.6	93	7.1	175	8.4	399	8.4
*Cx. pseudovishnui*	0	0.0	0	0.0	1	0.1	3	0.1	4	0.1
*Cx. quinquefasciatus*	13	1.8	30	5.5	22	1.7	106	5.1	176	3.7
*Cx. gelidus*	72	9.9	61	11.3	45	3.4	423	20.3	648	13.6
*Cx. fuscocephalus*	0	0.0	16	3.0	0	0.0	0	0.0	16	0.3
*Mansonia* spp.	0	0.0	19	3.5	0	0.0	0	0.0	19	0.4
*Ma. uniformis*	4	0.6	2	0.4	3	0.2	8	0.4	17	0.4
*Ma. annulifera*	5	0.7	3	0.6	16	1.2	6	0.3	33	0.7
*Ma. indiana*	0	0.0	0	0.0	0	0.0	1	0.0	1	0.0
*Armigeres* spp.	2	0.3	1	0.2	1	0.1	20	1.0	26	0.5
*Anopheles* spp.	32	4.4	112	20.7	69	5.3	36	1.7	254	5.3
Total	724	100	542	100	1309	100	2085	100	4660	100

Abbreviations: a, peripheral city district; b, peri-urban.

**Table 8:** Comparison of mosquitoes collected between pig owner bedroom and pig pen.

	Total Mosquitoes Collected	Number of Pig Farm	Mosquito Average Per Farm	95% CI	*p*-Value
In bedroom (indoor)	3039	179	17.0	6.70–27.2	0.202
In pig pen (outdoor)	4660	179	26.0	15.1–36.9	

**Table 9:** Results from univariable analysis showing the association between seropositivity of pigs and exposure variables.

Exposure Variable	Label	Total Test	Positive	Seroprevalence (95% CI)	OR (95% CI)	*p*-Value
Herd size	<10 pigs	98	70	71.4 (62.4–80.4)	Ref.	-
	10–29 pigs	256	228	89.1 (85.2–92.9)	3.26 (1.81–5.87)	<0.001
	30–59 pigs	189	178	94.2 (90.8–97.5)	6.47 (3.06–13.7)	<0.001
	≥60 pigs	93	87	93.5 (88.5–98.6)	5.80 (2.27–14.8)	<0.001
Breed	Crossbreed	387	326	84.2 (80.6–87.9)	Ref.	-
	Exotic	84	76	90.5 (84.1–96.8)	1.78 (0.82–3.87)	0.147
	Local	3	3	100	-	-
Age group	>6 months	44	29	65.9 (51.7–80.1)	Ref.	-
	2 months	56	55	98.2 (94.7–100)	28.4 (3.58–226)	0.002
	3 months	228	217	95.2 (92.4–98.0)	10.2 (4.28–24.3)	<0.001
	4 months	192	173	90.1 (85.9–94.3)	4.71 (2.15–10.3)	<0.001
	5 months	71	53	74.6 (64.4–84.9)	1.52 (0.67–3.46)	0.315
District	Dan Phuong	120	82	68.3 (60.0–76.7)	Ref.	-
	Chuong My	189	181	95.8 (92.9–98.6)	10.5 (4.68–23.5)	<0.001
	Bac Tu Liem	230	213	92.6 (89.2–96.0)	5.81 (3.10–10.9)	<0.001
	Ha Dong	97	87	89.7 (83.6–95.8)	4.03 (1.89–8.61)	<0.001

Abbreviations: OR, odds ratio; CI, confidence interval; Ref., Reference.

**Table 10:** Multivariable analysis of risk factors for pigs.

Exposure Variable	Categories	Coef.	OR	95% CI	*p*-Value
Herd size	<10 pigs	Ref.	Ref.		
	10–29 pigs	1.61	5.03	0.57–44.31	0.146
	30–59 pigs	2.39	10.94	0.97–122.8	0.053
	≥60 pigs	4.01	55.01	2.03–1491	0.017
Age group	>6 months	Ref.	Ref.		
	2 months	−1.33	0.26	0.006–10.7	0.481
	3 months	−2.15	0.12	0.003–4.75	0.256
	4 months	−3.42	0.03	0.0006–1.89	0.098
	5 months	−3.19	0.04	0.0004–3.76	0.166
District	Dan Phuong	Ref.	Ref.		
	Chuong My	2.97	19.4	1.19–315	0.037
	Bac Tu Liem	2.26	9.60	0.74–125	0.084
	Ha Dong	2.97	19.4	1.08–349	0.044
Constant		2.77	15.99	0.25–1019	0.191
		**Estimate**		**95% CI**	
Random effect of farm		9.80		4.63–20.75	

Abbreviations: Coef., Coefficients; Ref., Reference; OR, odds ratio; CI, confidence interval.

**Table 11:** Results from univariable analysis at farm level.

Exposure Variable	Label	Total HH Tested	HH Positive	OR (95%CI)	*p*-Value
District	Dan Phuong ^b^	41	32	Ref.	-
	Chuong My ^b^	53	53	-	-
	Bac Tu Liem ^a^	53	53	-	-
	Ha Dong ^a^	32	29	2.72 (0.67–11.0)	0.161
Herd size	<10 pigs	35	26	Ref.	-
	10–29 pigs	71	68	7.85 (1.97–31.3)	0.003
	30–59 pigs	47	47	-	
	≥60 pigs	26	26	-	-
Mosquito vector presence	No	22	21	Ref.	0.668
	Yes	157	146	0.63 (0.08–5.15)	
Family member not experienced with mosquito disease	No	167	156	Ref.	0.816
	Yes	12	11	0.78 (0.09–6.57)	
**Mosquito prevention practice by using:**					
Window/door screen	Yes	26	22	Ref.	0.07
	No	152	144	3.27 (0.91–11.8)	
Repellent	Yes	63	54	Ref.	0.008
	No	115	112	6.22 (1.62–23.9)	
Mosquito net	Yes	173	161	Ref.	-
	No	5	5	-	
Electric racket/portable electric trap	Yes	102	93	Ref.	0.211
	No	76	73	2.35 (0.61–9.01)	
Mosquito coil/Incense stick	Yes	59	54	Ref.	0.518
	No	119	112	1.48 (0.45–4.88)	
Long-sleeve clothes	Yes	64	55	Ref.	0.009
	No	114	111	6.05 (1.58–23.3)	
Lid covered on water tank	Yes	76	66	Ref.	0.01
	No	102	100	7.58 (1.16–25.7)	
Chemical/larvicide in water container	Yes	11	9	Ref.	0.14
	No	167	157	3.49 (0.66–18.3)	
Insecticides spraying	Yes	97	89	Ref.	0.386
	No	81	77	1.73 (0.50–5.97)	
Breeding site elimination	Yes	63	61	Ref.	0.178
	No	115	105	0.34 (0.07–1.62)	
Fish in water container	Yes	77	69	Ref.	0.102
	No	101	97	2.81 (0.81–9.71)	

Abbreviations: HH, Household; OR, odds ratio; CI, confidence interval; a, peripheral; b, peri-urban.

## Data Availability

All datasets supporting our findings are available from the corresponding author on reasonable request.
